# High-Resolution Melting System to Perform Multilocus Sequence Typing of *Campylobacter jejuni*

**DOI:** 10.1371/journal.pone.0016167

**Published:** 2011-01-24

**Authors:** Simon Lévesque, Sophie Michaud, Robert D. Arbeit, Eric H. Frost

**Affiliations:** 1 Department of Microbiology and Infectious Diseases, Faculté de Médecine et des Sciences de la Santé de l'Université de Sherbrooke, Sherbrooke, Québec, Canada; 2 Infectious Diseases Section, Tufts University School of Medicine, Boston, Massachusetts, United States of America; Institut de Pharmacologie et de Biologie Structurale, France

## Abstract

Multi-locus sequence typing (MLST) has emerged as the state-of-the-art method for resolving bacterial population genetics but it is expensive and time consuming. We evaluated the potential of high resolution melting (HRM) to identify known MLST alleles of *Campylobacter jejuni* at reduced cost and time. Each MLST locus was amplified in two or three sub fragments, which were analyzed by HRM. The approach was investigated using 47 *C. jejuni* isolates, previously characterized by classical MLST, representing isolates from diverse environmental, animal and clinical sources and including the six most prevalent sequence types (ST) and the most frequent alleles. HRM was then applied to a validation set of 84 additional *C. jejuni* isolates from chickens; 92% of the alleles were resolved in 35 hours of laboratory time and the cost of reagents per isolate was $20 compared with $100 for sequence-based typing. HRM has the potential to complement sequence-based methods for resolving SNPs and to facilitate a wide range of genotyping studies.

## Introduction

*Campylobacter jejuni* is the leading reported cause of bacterial gastroenteritis in developed countries [1]. The organisms colonize a range of hosts, including domestic animals and wild birds, and fecal shedding readily contaminates ground water [1]. While outbreaks are well documented, most clinical cases represent isolated, sporadic infections for which the source is rarely apparent. Consumption of contaminated food, especially poultry has been considered the most prevalent source [2]; however, recent studies implicate environmental water and unpasteurized milk as potentially important [3].

Multi-locus sequence typing (MLST), a genotyping system based on single-nucleotide polymorphisms (SNPs) of housekeeping genes, has emerged as the state-of-the-art method for resolving bacterial population genetics [4], [5]. A recently developed MLST system for *C. jejuni* [6] indicates that the species is genetically diverse, with a weakly clonal population structure, marked by frequent intra- and interspecies horizontal genetic exchange [6]–[8]. Some MLST-defined lineages of *C. jejuni* have been linked to a restricted geographical area [9] or to particular ecological niches, such as bathing beaches [7], water [10], wild birds [11], chickens, pigs, bovines or sheep [12]. Although MLST is highly reproducible, portable, and easy to interpret, it is complex and expensive to perform.

The development of fluorescent DNA binding dyes with improved saturation properties has allowed a more precise assessment of sequence variation based on the analysis of DNA melting curves. This technique, referred to as high resolution melting (HRM), can distinguish single base variation and then has the potential to identify SNPs without the burden of sequencing [13], [14]. After PCR amplification, amplicons are subjected to melting curves with a fluorescence monitoring of a saturating dye that does not inhibit PCR. This approach provides a simple, closed-tube, semi-automated and cost-effective method for detecting base substitutions and small insertions or deletions [15]. Merchant-Patel et al. [16] recently reported the application of HRM for typing the flagellin-encoding *flaA* gene of *Campylobacter jejuni*; their results demonstrated that the method is both accurate and easy to implement.

In this study, we describe the novel application of an HRM protocol optimized to perform MLST of *C. jejuni* isolates. Our goal was to resolve the *C. jejuni* sequence types as defined in the existing MLST database (http://pubmlst.org/campylobacter) at substantially lower cost than the conventional sequence-based method.

## Results

For all 47 isolates, successful amplifications were achieved across the 17 sub fragments spanning the seven MLST loci. Tables 1, 2, 3, 4, 5, 6, 7 list all SNPs included in this study. The SNP position in the fragment did not have a strong effect on the T_m_ separation, even if the SNP was near the amplification primer. Excluding *uncA*, about 90% of SNPs were transition mutations (T to C or C to T, A to G or G to A), but inversion mutations (G to C or C to G and A to T or T to A) were also readily detected.

**Table 1:** SNPs in *aspA* locus fragments.

Allele	SNPs position (5′ to 3′) in locus fragments^a^							
	9	45	84	-----174	-----279	342	414	476
*aspA*-1	T	G	G	G	C	C	T	C
*aspA*-2	T	G	A	A	T	C	C	T
*aspA*-4	C	A	G	G	C	T	T	C
*aspA*-7	T	G	A	A	T	C	T	T
*aspA*-8	T	G	G	G	T	C	C	T

a The numbering starts at the first nucleotide of each comparison fragment for each locus on the *C. jejuni* MLST database website. Numbers not underlined are in the left fragment, numbers with intermittent underlining are in the middle fragments and numbers with solid underling are in the right fragment.

**Table 2:** SNPs in *glnA* locus fragments.

Allele	SNPs position (5′ to 3′) in locus fragments^a^										
	12	33	45	-----108	-----112	-----132	-----202	-----267	369	384	465
*glnA*-1	G	A	A	A	C	A	A	C	C	T	A
*glnA*-2	G	A	A	G	C	A	A	C	C	T	A
*glnA*-4	G	G	A	G	T	A	G	T	T	C	G
*glnA*-7	A	A	A	G	C	T	A	C	T	T	A
*glnA*-17	G	G	G	G	T	A	A	C	C	T	A

a The numbering starts at the first nucleotide of each comparison fragment for each locus on the *C. jejuni* MLST database website. Numbers not underlined are in the left fragment, numbers with intermittent underlining are in the middle fragments and numbers with solid underling are in the right fragment.

**Table 3:** SNPs in *gltA* locus fragments.

Allele	SNPs position (5′ to 3′) in locus fragments^a^								
	12	39	200	201	207	294	320	348	396
*gltA*-1	A	C	T	G	C	C	G	A	A
*gltA*-2	G	T	T	G	T	C	A	A	A
*gltA*-3	A	T	C	G	C	T	A	G	A
*gltA*-5	A	C	T	G	C	C	A	A	A
*gltA*-10	A	T	T	C	C	T	A	A	G

a The numbering starts at the first nucleotide of each comparison fragment for each locus on the *C. jejuni* MLST database website. Numbers not underlined are in the left fragment and numbers with solid underling are in the right fragment.

**Table 4:** SNPs in *tkt* locus fragments.

Allele	SNPs position (5′ to 3′) in locus fragments^a^											
	12	21	72	117	138	141	162	174	189	297	330	435
*tkt*-1	C	C	T	C	C	T	A	A	C	C	T	C
*tkt*-3	C	C	T	C	C	T	A	A	C	C	C	C
*tkt*-7	T	T	A	C	A	C	A	G	T	T	C	T
*tkt*-9	T	T	A	T	C	T	G	G	T	T	C	T

a The numbering starts at the first nucleotide of each comparison fragment for each locus on the *C. jejuni* MLST database website. Numbers not underlined are in the left fragment and numbers with solid underling are in the right fragment.

**Table 5:** SNPs in *uncA* locus fragments.

Allele	SNPs position (5′ to 3′) in locus fragments^a^			
	3	-----189	-----234	375
*uncA*-1	T	C	G	C
*uncA*-3	C	C	G	T
*uncA*-5	C	T	G	C
*uncA*-6	C	C	G	C
*uncA*-17^b^				
*uncA*-105	T	C	A	C

a The numbering starts at the first nucleotide of each comparison fragment for each locus on the *C. jejuni* MLST database website. Numbers not underlined are in the left fragment, numbers with intermittent underlining are in the middle fragments and numbers with solid underling are in the right fragment.

b Left fragment: 12 SNPs; middle fragment: 29 SNPs; right fragment: 28 SNPs.

**Table 6:** SNPs in *glyA* locus fragments.

Allele	SNPs position (5′ to 3′) in locus fragments^a^																						
	3	42	51	57	114	120	129	136	138	198	208	213	237	259	264	267	285	286	303	309	312	320	504
*glyA*-2	T	C	C	T	T	A	C	C	T	C	G	C	A	A	C	A	A	C	T	T	G	C	C
*glyA*-3	C	T	T	C	T	A	C	C	T	T	A	T	A	G	T	G	A	T	C	T	A	C	T
*glyA*-4	T	C	C	T	C	G	G	T	A	T	A	T	A	G	C	G	G	C	C	C	A	T	C
*glyA*-53	T	T	T	C	T	A	C	C	T	T	A	T	G	G	T	G	A	T	C	T	A	C	T

a The numbering starts at the first nucleotide of each comparison fragment for each locus on the *C. jejuni* MLST database website. Numbers not underlined are in the left fragment and numbers with solid underling are in the right fragment.

**Table 7:** SNPs in *pgm* locus fragments.

Allele	SNPs position (5′ to 3′) in locus fragments^a^																									
	33	41	45	81	150	162	165	168	171	216	219	219	249	267	291	316	324	342	348	372	405	408	435	453	471	494
*pgm*-1	A	C	T	A	A	A	A	T	A	A	C	C	A	C	G	T	C	C	G	T	T	T	T	C	C	C
*pgm*-2	G	T	C	G	G	G	T	A	G	G	T	T	G	T	T	C	C	T	A	C	T	C	T	T	T	T
*pgm*-5	A	C	T	G	A	A	A	T	A	A	C	C	A	T	G	T	T	T	G	T	T	T	C	C	C	C
*pgm*-6	A	C	T	G	G	G	C	A	G	A	C	C	A	T	T	C	C	T	A	C	C	C	T	T	T	C
*pgm*-10	A	C	T	G	A	A	G	T	A	A	C	C	A	T	G	T	C	T	G	T	T	T	T	C	C	C
*pgm*-11	G	T	C	G	G	G	C	A	G	G	T	T	G	T	T	C	C	T	A	C	T	C	T	T	T	T

a The numbering starts at the first nucleotide of each comparison fragment for each locus on the *C. jejuni* MLST database website. Numbers not underlined are in the left fragment and numbers with solid underling are in the right fragment.

**Figure 1:**
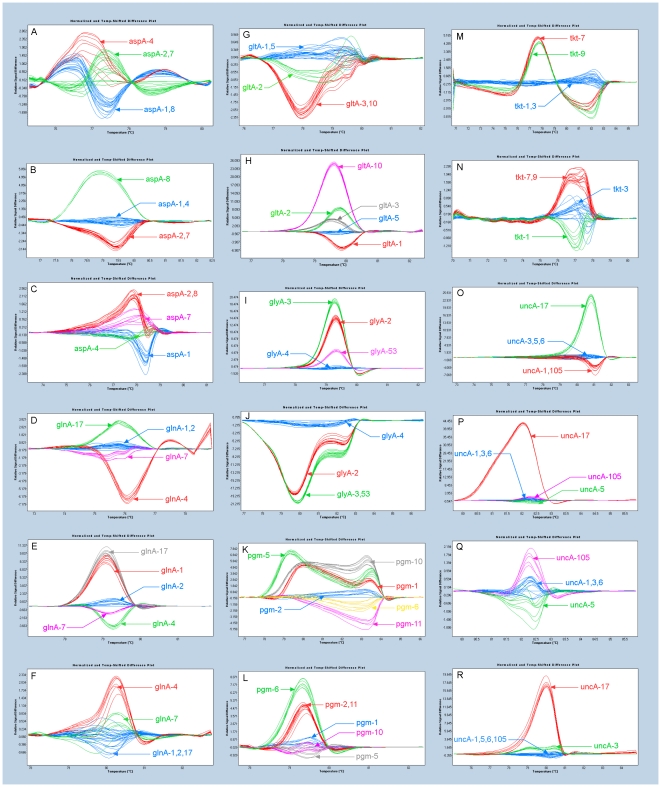
Difference plots for the normalized and temperature shifted melting curves for all locus fragments. **A:** asp left. **B:** asp middle. **C:** asp right. **D:** gln left. **E:** gln middle. **F:** gln right. **G:** glt left. **H:** glt right. **I:** gly left. **J:** gly right. **K:** pgm left. **L:** pgm right. **M:** tkt left. **N:** tkt right. **O:** unc left. **P:** unc middle. **Q:** unc middle without allele unc-17. **R:** unc right. Arrows link allele numbers with corresponding same color curves.

For each sub fragment, the expected 3 to 6 alleles were resolved by HRM as distinct difference plots (Figure 1). The reproducibility of the system was confirmed at multiple levels. The same DNA extracts were run in duplicate or triplicate wells of the same plate and in replicate wells across different runs. In addition, gene fragments representing the same MLST allele were amplified from DNA extracts of at least 6 different *C. jejuni* isolates. The HRM curves for the same DNA preparations or for the same sequences (SNPs) amplified from different isolates were readily grouped together; conversely, curves for different alleles could be consistently resolved.

Since each MLST locus was divided into two or three sub fragments for the HRM analysis, it was necessary to consider the HRM profiles for all of the sub fragments together in order to assign an MLST allele. For example, *aspA* was represented by five alleles and the locus was analyzed in three fragments (Table 1). The left fragment (199 bp) contained three SNPs; however, within this sub fragment, there were only three unique sequences – alleles *aspA*-1 and -8 were identical as were *aspA*-2 and -7. The middle fragment (197 bp) contained two SNPs generating three unique sequences; but in this region, *aspA*-1 and -4 were identical, as were *aspA*-2 and -7. Finally, the right fragment (247 bp) included 3 SNPs generating 4 unique sequences, with *aspA*-2 and -8 being identical. Within each sub fragment, the unique sequences had distinct HRM profiles (Figure 1A, 1B, and 1C). The combination of profiles across the three sub fragments resolved the five different alleles.

The *uncA* alleles in the demonstration set included *uncA*-17, which is derived from *C. coli* [6] and differs from the other *uncA* alleles by multiple SNPs, representing both transition and substitution mutations. Consequently, the HRM profile for each sub fragment of *uncA*-17 was highly divergent from the profiles for the other *uncA* alleles, with appreciably higher values for the relative signal difference (y axis, Figure 1O, P and R). For the middle sub fragment the other alleles were particularly difficult to resolve when *uncA-17* was present (Figure 1P), but readily distinguished when *uncA-17* was excluded (Figure 1Q).

To evaluate the relative efficiency and cost of performing MLST by HRM compared with conventional direct sequencing, we analyzed a confirmation set of 84 additional *C. jejuni* isolates from chickens. Using HRM, we resolved 92% of the MLST alleles. Moreover, the analysis required only 35 hours of laboratory time and reagents cost only $20 (Canadian) per isolate compared with $100 for sequence-based typing (data not shown).

## Discussion

MLST has emerged as the state-of-the-art method for studying bacterial population genetics. The MLST system for *C. jejuni* has been used in population studies of isolates from different geographical areas [17], from human and non-human sources [7], [9], as well as in molecular epidemiologic analyses of outbreaks [18], [19]. However performing MLST remains laborious and expensive. We have shown here that HRM can complement full MLST characterization of *C. jejuni* by identifying the most common alleles more rapidly and at lower cost.

HRM can resolve the SNPs that define the different alleles in the MLST system because two DNA amplicons that differ at even a single nucleotide will have different melting profiles. For the demonstration set of 47 diverse isolates, HRM resolved all 35 predicted alleles among the seven MLST genes. The differences in melting profiles among alleles varied with the number and type of SNPs as well as the gene fragment being amplified. For example, the profiles for *unc*-17, an allele which is known to come from *C. coli* [6], [8], [10], [11], [20], showed very strong differences in relative fluorescence signal and very sharp groupings (Figure 1O, P and Q). However, sub fragments where the alleles differed by only a single SNP often showed readily distinguished melting profiles (e.g., *aspA*-2, 7 and *aspA*-1, 8 in Figure 1A and *aspA*-7 and *aspA*-1 in Figure 1C). Even in instances where the relative fluorescence signal differences were quite small (0.8–3.0) and, consequently, the profiles less tightly clustered (Figure 1A, C and F), reliable interpretation was possible based on the differences in the overall profiles considered across the range of temperatures.

This strategy for typing *C. jejuni* isolates has many important advantages, but the single greatest benefit is the reduction in the total time and cost required. HRM requires neither agarose gel analysis, sequencing, nor sequence analysis. We estimate that the per isolate cost to perform MLST using HRM is 20–30% that of sequencing. This is achieved without compromising the portability of MLST since the existing nomenclature can be used. As of August 2010, the *C. jejuni* MLST database contained more than 4600 alleles among almost 10330 isolates. The 47 isolates in our study were drawn from the six major clonal complexes and included alleles whose frequency in the current database ranged from 40% and 68% (*pgm* and *uncA*, respectively). We were able to resolve most of these alleles using a single reference isolate for each of the six major sequences types. Distinguishing all known alleles might require additional reference isolates. However, an advantage of this HRM system is that any sequenced allele can be used as the reference profile. Our experience with the 84 *C. jejuni* isolates from chickens demonstrated that the system is particularly efficient when analyzing ecological niches with relatively few ST variations. Analyzing isolates from niches with more variation, novel niches, or from several niches simultaneously would be less efficient as it would require additional reference strains or sequencing more samples, but would still be less expensive than sequencing of all genes.

Obviously, an HRM system cannot replace sequence-based MLST. If a previously unidentified melting profile is encountered, it is necessary to revert to sequencing; however, once identified, the new profile can be used for reference in subsequent HRM analyses. If the sequence proves to be a new allele, it can be submitted to the database.

At a technical level, HRM can be performed using cyclers that accept 384-well plates, permitting high-throughput studies. Because HRM compares amplicons from independent PCR reactions, it is essential to standardize the quantity of DNA used in order to minimize reaction-to-reaction variability. We observed that variation in DNA quantity or quality could shift amplification curves; such offsets have been previously observed to compromise the HRM groupings [21].

HRM can be readily applied to a wide range of genetic analyses that involve detection of a single SNP or a signature allele representing a specific set of SNPs. Examples in microbiology include studies requiring the identification of particular clonal complexes, sequence types or individual mutations. By selecting the locus amplified and the reference standard for the HRM system based on the objective of the study, this approach can be applied to questions in pathogenesis, ecology, epidemiology and antibiotic resistance. As just one example, the NAP1/027 epidemic strain of *C. difficile* belongs to MLST type 35 [22]. Identifying a signature allelic profile could serve as a rapid shortcut for preliminary strain detection [23], [ 24], minimizing the challenges and effort associated with PFGE or sequencing. Analogous situations arise in numerous studies across all levels of biology, from resistance mutations in viruses to human alleles associated with clinical disease.

In summary, we have demonstrated that by analyzing multiple loci concurrently HRM technology can resolve the SNPs that are the basis of MLST. In our studies of >120 *C. jejuni* isolates from diverse geographical sources and representing diverse genotypes, the HRM results were consistent with sequencing and thus could be expressed using the existing MLST nomenclature, but were obtained with greater speed, less effort and at lower cost. HRM has the potential to complement classical sequence-based methods and facilitate a wide range of genotyping studies.

## Materials and Methods

### Isolates

Table 8 lists the source, MLST alleles, sequence type and clonal complex of 47 *C. jejuni* isolates used in this study; all have been previously reported [10] and analyzed by the standard MLST protocol [7]. Isolates were selected to represent diverse sources and to include the six most prevalent sequence types (ST) and most frequent alleles for each locus.

**Table 8:** *C. jejuni* isolates used in the study.

Isolate	Source	*aspA*	*glnA*	*gltA*	*glyA*	*pgm*	*tkt*	*uncA*	ST^a^	CC^b^
001A-0058	Human	2	1	1	3	2	1	5	21	21
001A-0078	Human	2	1	1	3	2	1	5		
001B-0003	Chicken	2	1	1	3	2	1	5		
001B-0035	Chicken	2	1	1	3	2	1	5		
001B-0046	Chicken	2	1	1	3	2	1	5		
006A-0001	Raw milk	2	1	1	3	2	1	5		
006A-0004	Raw milk	2	1	1	3	2	1	5		
007A-0018	Water	2	1	1	3	2	1	5		
007A-0031	Water	2	1	1	3	2	1	5		
001A-0059	Human	4	7	10	4	1	7	1	45	45
001A-0060	Human	4	7	10	4	1	7	1		
001B-0010	Chicken	4	7	10	4	1	7	1		
001B-0011	Chicken	4	7	10	4	1	7	1		
001B-0024	Chicken	4	7	10	4	1	7	1		
007A-0023	Water	4	7	10	4	1	7	1		
007A-0030	Water	4	7	10	4	1	7	1		
007A-0032	Water	4	7	10	4	1	7	1		
001A-0005	Human	7	17	5	2	10	3	6	353	353
001A-0016	Human	7	17	5	2	10	3	6		
001A-0085	Human	7	17	5	2	10	3	6		
001A-0259	Human	7	17	5	2	10	3	6		
001A-0263	Human	7	17	5	2	10	3	6		
001A-0273	Human	7	17	5	2	10	3	6		
001A-0274	Human	7	17	5	2	10	3	6		
001B-0008	Chicken	7	17	5	2	10	3	6		
001A-0162	Human	1	4	2	2	6	3	17	61	61
001A-0163	Human	1	4	2	2	6	3	17		
001A-0166	Human	1	4	2	2	6	3	17		
001A-0238	Human	1	4	2	2	6	3	17		
006A-0014	Raw milk	1	4	2	2	6	3	17		
006A-0020	Raw milk	1	4	2	2	6	3	17		
006A-0026	Raw milk	1	4	2	2	6	3	17		
006A-0028	Raw milk	1	4	2	2	6	3	17		
001A-0064	Human	1	2	3	4	5	9	3	42	42
001A-0084	Human	1	2	3	4	5	9	3		
001A-0088	Human	1	2	3	4	5	9	3		
001A-0168	Human	1	2	3	4	5	9	3		
001B-0009	Chicken	1	2	3	4	5	9	3		
001B-0012	Chicken	1	2	3	4	5	9	3		
001B-0052	Chicken	1	2	3	4	5	9	3		
006A-0053	Raw milk	1	2	3	4	5	9	3		
001A-0287	Human	8	2	5	53	11	3	105	1212	1212
001A-0289	Human	8	2	5	53	11	3	105		
001B-0029	Chicken	8	2	5	53	11	3	105		
001B-0055	Chicken	8	2	5	53	11	3	105		
001B-0056	Chicken	8	2	5	53	11	3	105		
001B-0057	Chicken	8	2	5	53	11	3	105		

a ST; sequence type.

b CC; clonal complex.

### DNA extraction

All *C. jejuni* isolates were grown on 5% (vol/vol) defibrinated sheep blood TSA (Oxoid Inc., Nepean, On) in a micro aerobic atmosphere at 42°C for 24–48 h. Isolated colonies were used to inoculate Mueller-Hinton broth (Oxoid Inc., Nepean, On), grown to 0.5 McFarland standard density, 0.5 ml of the broth transferred to a microfuge tube, centrifuged at 13000 rpm for 10 minutes and the supernatant discarded. Genomic DNA was extracted from the pellet by adding 10 µl of NaOH 0.5 N. After 5 minutes, 10 µl of Tris 1 M pH 8.0 and 980 µl of sterile distilled water were added. DNA extracts were stored at −20°C. DNA concentration was measured using a NanoVue spectrophotometer (GE Healthcare Life Science, Piscataway, NJ, USA).

### Primer design

The fragments for the seven loci in the MLST system (402 to 507 bp) are longer than the maximum that can be efficiently analyzed by HRM (100 to 300 bp) [13]. Consequently, for each locus two or three sub fragments were analyzed to provide adequate resolution of the known alleles. Oligonucleotide primers used are listed in Table 9. In the majority of cases, the 3′ end (for forward primers of left locus fragments) and the 5′ end (for reverse primers of right locus fragments) were the last nucleotides before/after the comparison fragment for each locus on the *C. jejuni* MLST database website. In four cases (GLN HRM F7, TKT HRM F1, TKT HRM R2, UNC HRM F6) the primer was upstream or downstream from the comparison fragment by −8, −4, +4 and −6 nucleotides, respectively. One primer (GLY HRM F3) included the first nucleotide of the comparison fragment. Internal primers overlapped each other to cover the entire sequence. Primers were synthesized by Integrated DNA Technologies (Coralville, Iowa, USA) and used without further purification.

**Table 9:** Oligonucleotide primers used in the study.

Locus	Locus fragment	Forward (5′ to 3′)		Reverse (5′ to 3′)		Amplicon size (bp)
*aspA*	asp left	ASP HRM F3	GTG AAT TTA AAA CTT TTG CCG TA	ASP HRM R6	TCG ATC AAA TCC TCA GCC ACA GTA	199
	asp middle	ASP HRM F5	TAA GAG AAG TGA CAG GTT TTG AAT	ASP HRM R7	GGA AGA TTA ATC TCA TTA AGA CCA CAT T	187
	asp right	ASP HRM F7	GAC TTA AGA CTT TTA AGT AGT GGT CC	ASP HRM R4	GCA TTA CAA CAG AAT TAA ATA AGC TAT ATG C	247
*glnA*	gln left	GLN HRM F6	AAC CTG ATG CTC AAA GTG C	GLN HRM R5	CAT TTT TCA TAC ATT TGT CCT TTG	106
	gln middle	GLN HRM F7	CTA TCA TAG TAT TTT GTG ATG TGT ATG	GLN HRM R4	CTA AAG AAT CAA TTG GCT GAA CTG G	318
	gln right	GLN HRM F4	CTG GAC ACA GGC CAA GAA ACA AAG GTG	GLN HRM R2	GAG CTA CCA TTT TTA CAA CAT ATT TAT AAA TTT G	231
*gltA*	glt left	GLT HRM F1	CGC GTC TTG AAG CAT TTC GTT AT	GLT HRM R1	CCA CTA TAG TAG GGA TTT TAG CTA C	225
	glt right	GLT HRM F2	GAA TAT ATG GAA ATG GCA GCT AG	GLT HRM R2	GCA TGA GTT GAA CCC ACA GC	272
*glyA*	gly left	GLY HRM F3	GAT AAA ATT TTA GGA ATG GAT TTA AGT CAT G	GLY HRM R1	CAC AAC AAG ACC TGC AAT ATG	288
	gly right	GLY HRM F2	GCC TAT CTT TTT GCT GAT ATA GCA	GLY HRM R2	AAA ACA TTA GCT AAA ACT TGA GC	317
*pgm*	pgm left	PGM HRM F1	GAA GTT ATA GTA ATG AGT GAT AAA CCT AAT G	PGM HRM R1	TTT AAA GCA CCA TTA CTC ATT ATA GT	275
	pgm right	PGM HRM F4	GGT AAA TTA CAA TCA AGT GTT GTG GC	PGM HRM R3	CTT TTT TTT CTG CAA TTT TAA G	328
*tkt*	tkt left	TKT HRM F1	CAT GCA AGT GCT TTG CTT TAT AGT	TKT HRM R1	CCC ATC TCC GCA AAG ACA A	261
	tkt right	TKT HRM F2	GCT AGG CAG TGA TTT AAT CGA TCA	TKT HRM R2	GAT GAT AAG ACA AGG TTT TGT GGA	304
*uncA*	unc left	UNC HRM F6	GGT GCT ATG GAA TAT ACT ATT GTT G	UNC HRM R3	GAC ATT TCG CGA TAA GCT ACA GC	176
	unc middle	UNC HRM F7	GTT TAT GAT GAT TTG AGC AAG C	UNC HRM R4	GTT GGA ATA TAA GCA GAA ACA TCT CC	221
	unc right	UNC HRM F8	GGT GCT GGT TCT TTG ACG GCA TTG	UNC HRM R2	GTG CAA AAG CTT GAA GCT CTC TA	265

### PCR and HRM analysis

Real-time PCR cycling was performed in a 96-well plate on a LightCycler® 480 II real-time PCR system (Roche). Each plate must contain at least two reference isolates for each allele that would be identified on the plate together with the unknown samples. The reaction was performed in a 15 µl PCR mix containing 1X LightCycler® 480 High Resolution Melting Master Kit (Roche), 3.5 mM MgCl_2_, 0.5 µM of each primer and between 10 and 20 ng of DNA The amplification protocol consisted of a first denaturation step at 95°C [5 min], 45 cycles of denaturation at 95°C [10 s], annealing at 55°C [30 s], and extension at 72°C [30 s]. The HRM step consisted of a first denaturation step at 95°C [1 min], followed by a renaturation step at 40°C [1 min]. Melting curves were generated by ramping from 70°C to 95°C at 0.02°C/sec, 25 acquisitions/°C.

**Figure 2:**
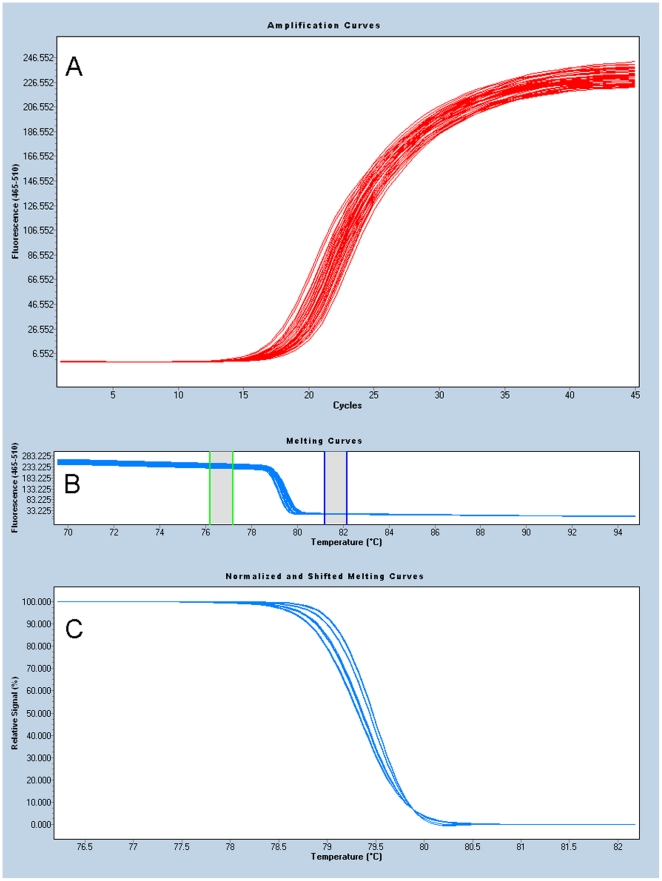
Data preparation for HRM. **A**: Amplification curves for *tkt* right fragment for 96 isolates. **B**: Normalization of raw melting curve data. Green box: pre-melt (initial fluorescence). Blue box: post-melt (final fluorescence). **C**: Normalized and shifted melting curves.

During amplification, fluorescence data were normalized and then plotted using the automated grouping functionality provided by the LightCycler® 480 II Gene Scanning Software version 1.5.0.39 and by manual editing. Figure 2A shows the compilation of curves representing successful amplification of the left fragment of *gly* for 96 isolates. All curves reached a similar plateau height and, as per manufacture's recommendations, the mean cycle number at which fluorescence exceeded background (referred to as the crossing point or cycle threshold) was <30 with a range of less than 7 across all samples. Reactions that did not meet these criteria were discarded and the fragment amplified again in a subsequent run. The software automatically analyzed the raw melting curve data and set the pre-melt (initial fluorescence) and post-melt (final fluorescence) signals of all samples to uniform values (Figure 2B); occasionally, manual adjustments were made to optimize group separation. Next, the software shifted the normalized curves along the temperature axis to equalize the point at which the dsDNA in each sample becomes completely denatured (temperature shift, Figure 2C). For each locus, the default of 5 was used as the threshold value in the temperature shift step. In the final step each shifted, normalized curve is plotted (difference plot, Figure 1I) as the difference relative to an arbitrarily chosen reference curve among the samples analyzed on the plate, usually one of the known reference isolates. The software groups together similar curves according to an adjustable sensitivity value. In these displays (Figure 1A to 1R) the differences between melting curve profiles for different alleles are readily appreciated. Curves not grouped with one of the reference isolates would have to be run subsequently with other reference isolates containing the allele or sequenced. If the reference isolates were not grouped together correctly, the run would be repeated.
